# The Immunology of Mammary Gland of Dairy Ruminants between Healthy and Inflammatory Conditions

**DOI:** 10.1155/2014/659801

**Published:** 2014-11-10

**Authors:** Mohamed Ezzat Alnakip, Marcos Quintela-Baluja, Karola Böhme, Inmaculada Fernández-No, Sonia Caamaño-Antelo, Pillar Calo-Mata, Jorge Barros-Velázquez

**Affiliations:** ^1^Department of Analytical Chemistry, Nutrition and Food Science, School of Veterinary Sciences/College of Biotechnology, University of Santiago de Compostela, Campus Lugo, Rúa Carballo Calero, 27002 Lugo, Spain; ^2^Food Control Department, Dairy Division, Faculty of Veterinary Medicine, Zagazig University, Zagazig, Al Sharkia 44519, Egypt

## Abstract

The health of dairy animals, particularly the milk-producing mammary glands, is essential to the dairy industry because of the crucial hygienic and economic aspects of ensuring production of high quality milk. Due to its high prevalence, mastitis is considered the most important threat to dairy industry, due to its impacts on animal health and milk production and thus on economic benefits. The MG is protected by several defence mechanisms that prevent microbial penetration and surveillance. However, several factors can attenuate the host immune response (IR), and the possession of various virulence and resistance factors by different mastitis-causing microorganisms greatly limits immune defences and promotes establishment of intramammary infections (IMIs). A comprehensive understanding of MG immunity in both healthy and inflammatory conditions will be an important key to understand the nature of IMIs caused by specific pathogens and greatly contributes to the development of effective control methods and appropriate detection techniques. Consequently, this review aims to provide a detailed overview of antimicrobial defences in the MG under healthy and inflammatory conditions. In this sense, we will focus on pathogen-dependent variations in IRs mounted by the host during IMI and discuss the potential ramifications of these variations.

## 1. Introduction

The udder is the milk-producing organ of dairy animals; hence, for optimal production, it should be healthy. Mastitis is the inflammatory response of the mammary gland (MG) tissue to physiological and metabolic changes, traumas, and allergies and, most frequently, to injuries caused by various microorganisms. Mastitis is considered the utmost threat to the dairy industry from three perspectives: economic, hygienic, and legal (EU Directive 46/92, modified by Directive 71/94). The intramammary inflammation (IMI), accompanied by immunological and pathological changes in the MG tissue, occurs at different degrees of intensities and results in a wide range of consequences regarding physical, chemical, and often microbiological alterations of secreted milk.

A wide spectrum of microorganisms, including fungi, yeast, algae,* Chlamydia*, and viruses, have been incriminated in causing mastitis, but bacteria remain the principle causative agents of such complex [1, 2]. The major bacterial mastitis pathogens (*Staphylococcus aureus*,* Streptococcus agalactiae*,* S. uberis*,* S. dysgalactiae*, and coliforms) are most often responsible for clinical mastitis (CM). Meanwhile, minor pathogens (coagulase-negative staphylococci “CNS”; streptococci other than* S. agalactiae*,* S. uberis*, and* S. dysgalactiae*;* Corynebacterium* spp.;* Pseudomonas* spp.;* Serratia* spp.;* Proteus* spp.;* Pasteurella* spp.;* Listeria* spp.;* Leptospira* spp.;* Yersinia* spp.;* Enterobacter* spp.;* Brucella* spp.; and* Mycobacterium* spp.) are typically associated with subclinical mastitis (SCM) or sometimes associate clinical IMIs [2]. With the exception of a few pathogens that can invade via the blood stream (e.g.,* Brucella abortus *or* Mycobacterium bovis*), infection of the MG occurs by ascension through the ductus papillaris, the only opening of the udder to the outside world [3], and the pathogens pass to find an environment that is warm, moist, and nutrient-rich and thus suitable for rapid growth and multiplication. To establish a successful infection after traversing the teat end opening, bacteria must combat the antimicrobial activities of the teat and MG microenvironments [4].

Adequate immune functions are essential for host defence against IMIs. MG immunity depends on the complex combination and coordination of nonspecific and specific protective elements, including the anatomical features of the gland as well as cellular and humoral defence components [5]. Nevertheless, MG immune defence varies over different stages of lactation in dairy animals and is typically depressed with exposure to stress and around drying-off and parturition, thus increasing susceptibility to mastitis [6, 7]. However, a considerable body of evidence has accumulated suggesting that mastitis is a multifactorial complex, and several management and environmental factors must interact to increase host exposure to mastitis pathogens, reduce the natural resistance of animals to disease, or aid pathogens in gaining entrance to the MG environment to cause infection [2, 4].

## 2. The MG Immune Defence System and Its Related Components

### 2.1. Teat Skin and Teat Canal Defence System (Structural Defence)

The primary defence mechanism of the MG is represented structurally in the teat canal [3, 8, 9], which acts as both a physical barrier and a source of antimicrobial substances. The physical barrier is provided by the smooth muscle sphincter surrounding the teat canal, which prevents escape of milk and constitutes a barricade against the entry of different pathogens by maintaining tight closure [2, 3, 9]. The antimicrobial defence, on the other hand, comprises several components. Normally, the healthy teat skin is coated with a protective mantle of fatty acids (FAs) that slow the growth of bacterial pathogens [2]. Additionally, the stratified squamous epithelium of the teat duct produces keratin, a waxy material lining the teat canal, which traps invading bacteria and hinders the migration of microorganisms into the gland cistern. Keratin also contains antimicrobial agents that assist in combating infection [9]. This keratin is composed of (I) bacteriostatic FAs of both esterified and nonesterified types, such as lauric, myristic, palmitoleic, and linolenic acids and (II) fibrous proteins, which bind electrostatically to microorganisms, altering the cell wall and rendering it more susceptible to osmotic pressure changes and, thus, to lysis and death [9]. Additionally, these cationic proteins were found to have an inhibitory effect against some pathogens as* Staph. aureus *and* S. agalactiae* [10], which was equal to that of proteins isolated from bovine neutrophils [11]. The lipid content and composition of teat duct keratin have been shown to vary throughout the milking process [12], between lactating and dry dairy animals [13], and according to the severity of IMI. SCM was found to not affect the lipid content of teat duct keratin, while CM was shown to be associated either with significantly higher levels of total lipids [14] or with similar lipid composition of uninfected quarters [15]. Additionally, the free FAs in milk from clinical quarters contained fewer short-chain FAs, whereas polyunsaturated FAs were significantly higher [14]. Recently, sentinel functions for the teat towards invading pathogens have been documented, as the teat canal tissue responded rapidly and intensely, with both expression of several Toll-like receptors (TLRs) and production of cytokines and antimicrobial peptides [16, 17].

Damage of keratin, perhaps as a result of incorrect intramammary therapy infusion [18] or by faulty machine milking [19], has been reported to increase susceptibility of the teat canal to bacterial invasion and colonisation [20]. However, the antimicrobial effectiveness of keratin is limited [9, 21] and, despite the potent physical and chemical protection in the teat canal, there are several ways by which bacteria can penetrate the teat canal and cause IMI, so much so that a number of pathogens are able to colonize the teat canal for prolonged periods, such as* Corynebacterium bovis*, or CNS. The survival for several days of* Staph*.* aureus* deposited a few mm inside the teat canal has also been demonstrated [22–24]. Also, during milking, it is common for keratin to be flushed out with distention of the teat canal [25]. Because the sphincter takes approximately 2 h to regain its contracted position, there is a chance for outside pathogens to enter the teat canal, causing trauma and damage to the keratin or mucous membranes lining the teat sinus [2, 21]. Additionally, during mechanical milking, microorganisms present at the teat end may be propelled into or through the teat duct into the cistern. This mechanism is considered the chief mechanism behind the spreading of contagious mastitis pathogens [26].

### 2.2. Innate and Adaptive (Acquired) Immunity

The MG is normally protected by both innate and adaptive immune responses (IRs), which coordinate and operate together to provide an optimal defence against infections. The IRs also facilitate the constitutive or acute transient presence of a wide range of immune-related components in milk [5]. The adaptive immune system (AIS) responds more robustly to threats to which it has previously been exposed [27]; however, it is slow to respond to novel threats [33]. In contrast, the innate immune system (IIS) is the first line of defence against pathogens once they have penetrated the physical barrier of the teat canal and before the AIS comes into play, and it evolves into a highly effective host defence [33, 34]. This process is mediated via several intracellular signal transduction cascades that trigger an acute upregulation of several innate immune components including different leukocytes, adhesion molecules, and cytokines [35–37].

#### 2.2.1. The IIS and Its Components

Innate immunity plays a vital role in protecting the MG against infection [38]. The two most critical components of host innate immunity are pathogen recognition (PR) and the ability to mount a proinflammatory response, a complex interaction of cellular and molecular processes aimed at detecting and subsequently eliminating harmful pathogens [25, 34]. A wide variety of components linked to the innate IR (IIR) have been identified in milk, including cellular defence components [e.g., leukocytes], components contributing to humoral defence [e.g., complement system (CS), immune-modulating factors (pro- and anti-inflammatory cytokines), lactoferrin (LF), transferrin (TF), lysozyme (LZ), and components of the lactoperoxidase/myeloperoxidase systems], oligosaccharides, gangliosides, reactive oxygen species (ROS), acute phase proteins (APPs) (e.g., haptoglobin and serum amyloid A), ribonucleases, and a wide range of antimicrobial peptides and proteins. Many of these components originate from specialised cells that traffic to the MG [33, 34]. The ability of the IIS to recognise and respond to a broad spectrum of pathogens that may or may not have been previously encountered, combined with the speed in mounting a proinflammatory response following initial PR, greatly contributes to the host's ability to control invading pathogens [37]. Below, there is a detailed overview of the roles and mechanisms of action of some innate immune factors.

(*1) Cellular Defence Systems and Roles of Different Immune Cells (Leukocytes).* The viable leukocytes inside the MG offer some degree of cellular protection against microbial invasion through their ability to recognise microorganisms and induce a rapid inflammatory response in an attempt to resolve the IMI immediately. Thus, MG-resident leukocytes likely provide a surveillance function in the uninfected gland. Also, these cells may aid in the restructuring of the MG that occurs during involution (i.e., apoptosis) [39]. In addition to microbicidal functions of phagocytosis, MG leukocytes secrete a variety of immune-related components into milk including cytokines, chemokines, ROS, and antimicrobial proteins and peptides (LF, defensins, and cathelicidins) [40]. Leukocytes also assist in the repair of damaged tissue caused by shedding and renewal processes [41]. Despite the presence of considerable numbers of immune cells in the MG environment, it has been suggested that the MG is immune-compromised when compared to the rest of the body [42]. Moreover, the activities of all types of leukocytes in milk have been shown to be reduced compared to those in blood [28, 43, 44]. The migration of immune cells during IMI plus desquamation of MG epithelia results in an increase of somatic cell count (SCC) accompanied with decreased milk production according to the severity of the process [1, 45].

*Neutrophils.* Polymorphonuclear neutrophils (PMNs) constitute the second line of the IIS against IMI. Even under healthy conditions, PMNs are permanently present inside the MG environment, and nursing or milking stimuli accompanied with milk removal were found to induce directed migration of fresh PMN into mammary tissue [46]. Bovine neutrophils cross the MG epithelium by diapedesis without causing epithelial cell damage [47] unless the migration is extensive, in which case both mechanical and chemical damage are possible [48]. The neutrophil's multilobulated nucleus allows for easy and rapid migration between endothelial cells, thus arriving as the first recruited immune cell to sites of infection [49]. Because only small numbers of mature PMNs are stored in the bone marrow, the number of immature neutrophils in circulation increases as a result of mobilisation into circulation during inflammatory conditions. Thus, the number of circulating mature neutrophils negatively correlates with severity of mastitis [6]. Several important functions are not fully developed in immature neutrophils, including those pertaining to phagocytosis, intracellular killing, and chemotaxis [50].

Neutrophils are delineated by a plasma membrane that has a number of functionally important receptors. These include L-selectin and *β*2-integrin adhesion molecules, which promote the binding of PMNs to endothelial cells and facilitate their migration to infected foci [39, 49, 51]. Membrane receptors for the Fc portion of the IgG2 and IgM classes of Igs and for complement components C3b and iC3b are necessary for the phagocytosis of invading bacteria [52, 53]. The activation of C3b regions on bacterial surfaces after binding with Abs promotes phagocytosis and binding to CR1 and CR3 receptors on the PMN surface [52]. Additionally, lectin-carbohydrate receptors found on neutrophil cell membranes can recognise carbohydrate-rich fimbriae of* Escherichia coli* in the absence of specific opsonins [49, 54], resulting in a process referred to as nonopsonic phagocytosis [54].

The primary function of PMNs to engulf, phagocytose, and destroy foreign materials, including invading bacteria, occurs via two parallel systems. The first is an oxygen-dependent (respiratory burst) system that includes the production of hydroxyl and oxygen radicals. The second is an oxygen-independent system that relies on several oxygen-independent reactants such as peroxidases, LZs, hydrolytic enzymes, and LF [1, 41]. In addition to phagocytic activity, PMNs also contribute to the modulation of vascular permeability and release several inflammatory mediators that play crucial roles in the coordination of innate and adaptive immune components [55]. Furthermore, the intracellular granules of PMNs contain several bactericidal peptides including defensins, enzymes (e.g., myeloperoxidase), and neutral and acidic proteases (e.g., elastase; cathepsin types B, D, and G, procathepsins) [56–58], which can kill a variety of mastitis pathogens. Such proteases as well as plasmin are known to permit the chemotaxis of cells in the site of inflammation and are involved in the limitation in time of the IR (e.g., by the cleavage of some cytokines such as IL-2, IL-6, and IL-8) [59]. The exposure of PMNs to cytokines and chemoattractants causes rapid mobilization of azurophil granules (containing elastase and cathepsin G mainly) to the cell surface [60]. Unfortunately, the released oxidants and proteases lack specificity. In bovines, PMNs can wrongly phagocytose milk fat globules [61], and their proteases can degrade milk casein (caseinolysis), leading to putrefaction of milk, and, together with their released hydroxyl radicals, can damage the MG epithelium which contributes to the decreased synthetic activity of the MG during IMI [62]. Once PMNs perform their tasks, they undergo apoptosis, or programmed cell death, and are removed by macrophages [63, 64].

*Macrophages.* Macrophages are produced from blood monocytes that differentiate within MG tissues. They constitute the predominant cell type found in milk and tissues of both healthy involuted and lactating BMGs [43, 65, 66]. In contrast to neutrophils, macrophages have large horseshoe-shaped nuclei that make their migration between endothelial cells more difficult [49]. Macrophages facilitate both innate and acquired IRs through performing several specific and nonspecific functions. Macrophages contribute to induction of specific local IRs through antigen (Ag) processing and presentation to lymphocytes in association with MHC class II Ags [45, 67–69]. Similar to PMNs, macrophages can perform a variety of nonspecific functions including ingestion and phagocytosis of foreign particles, including some invading bacteria (e.g.,* Staph. aureus*) [70], and destroying them with proteases and ROS [66, 70, 71]. Additionally, they can ingest cellular debris and accumulated milk components in involuting MGs [29]. The phagocytic activity of macrophages can be increased in the presence of opsonic Abs for specific pathogen [72]. In cattle, MG macrophages bear receptors for IgG1 and IgG2 [73]. Unlike neutrophils, macrophages possess fewer Fc receptors, which decrease their phagocytic capacity [7]. MG macrophages are considered less effective at phagocytosis compared to blood monocytes because of indiscriminate ingestion of milk components as well as the fact that macrophage proteases can also contribute to damage of MG epithelium [62, 70, 74]. A failure of efficient killing of some mastitis pathogens (e.g.,* S. uberis*) after engulfing and even increased intracellular multiplication of* S. uberis* as well as lesser stimulatory responses by IFN-*γ* to release TNF-*α* and bactericidal products compared to blood monocytes have been also reported [70]. However, it has been demonstrated that the bactericidal activity of MG macrophages can vary according to MG secretion, and dry-off secretion macrophages exerted higher bactericidal activities than lactational macrophages [70]. Therefore, the ability of macrophages to secrete substances that augment local inflammatory processes, thereby inducing the migration and bactericidal activities of neutrophils, is believed to be of greater importance to nonspecific defence of the MG than their function as professional phagocytes [7, 67, 70, 75].

**Figure 1:**
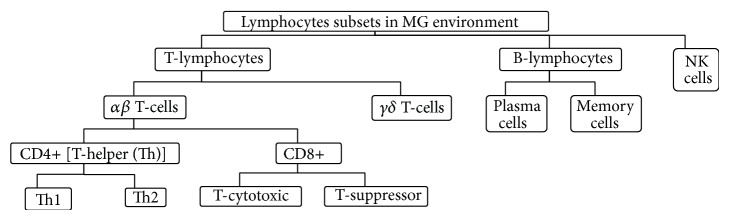
Different subsets of MG lymphocytes [27–32].

*Lymphocytes.* Lymphocytes are a type of immune cells that mediate specific responses to pathogens. Lymphocytes recognise a variety of antigenic structures via membrane receptors, which define their specificity, diversity, and memory characteristics. T- and B-lymphocytes and natural killer (NK) cells are distinct lymphocyte subsets that operate in the MG (Figure 1), although they differ in function and protein products [30]. During IMI, preferential trafficking of certain lymphocyte subpopulations to specific mammary tissue foci occurs [28, 76] and marked changes in milk lymphocyte count and composition during IMIs have been reported [45]. Once activated, lymphocytes can regulate both specific and nonspecific IRs [77]. Additionally, the AIS response is mainly mediated by memory lymphocytes, which respond quickly to threats to which they have previously been exposed [33]. It must be mentioned that the presence of specific lymphocyte subsets can affect the total lymphocyte function and even the whole IR. For example, the activation of CD8+ T-cells during certain bacterial IMIs, such as* Staph. aureus*, can suppress important host IRs and predispose to chronic pattern of IMI [78, 79]. Unfortunately, the exact roles of lymphocytes during IMI and their subsets are complex and are not fully defined. Even in healthy MGs, the composition of the lymphocyte population varies during the lactation cycle [28, 76, 80]; the consequences to MG immunity are still not fully understood. Additionally, MG lymphocytes exhibit hyporesponsiveness to mitogenic, antigenic, and allogeneic stimuli compared to blood lymphocytes, possibly due to the presence of distinct lymphocyte subsets, high proportion of memory T-lymphocytes present in the MG [28], and/or less efficient presentation of Ags by Ag-presenting MG cells [6].

In healthy BMGs, *αβ* T-cells prevail in both MG secretions and parenchyma and predominantly exhibit the CD8+ phenotype, which is in contrast to the blood, where CD4+ cells are the predominant T-cell subset [77]. Therefore, the ratio of CD4+/CD8+ T-cells is lower in milk than in blood. CD4+ (T-helper) cells produce a variety of immunoregulatory cytokines following Ag-recognition with MHC class II molecules; and are being memory cells following Ag-recognition [4, 7, 27, 81]. On the other hand, it is well established that CD8+ cells can exert either cytotoxic or suppressor functions. In coordination with major histocompatibility complex (MHC) class I molecules, cytotoxic T-cells recognise and eliminate altered self-cells via Ag presentation, thus being more specific than NK cells. However, it has been suggested that their removal of damaged mammary epithelium could enhance the susceptibility of MG to infection [28]. Suppressor T-cells are thought to play roles in control or modulation of the MGIR [76]. However, the immunoregulatory roles of CD8+ cells are also greatly dependant on lactation stage. Cells obtained from midlactation dairy cattle exhibited cytotoxic activity and mainly expressed interferon-*γ* (IFN-*γ*), whereas CD8+ lymphocytes obtained during the postpartum period exhibited no cytotoxic activity and mainly expressed interleukin 4 (IL-4) [76].

Ruminants bear greater levels of *γδ* T-lymphocytes in secretions and parenchyma of MG relative to blood [82]. There are indications that *γδ* T-cells can mediate cytotoxicity, similar to NK cells, with variable involvement of MHC molecules; thus, they may be able to destroy altered epithelial cells [83, 84]. *γδ* T-lymphocytes preferentially migrate to particular epithelial surfaces and do not exhibit extensive recirculation [85]. Thus, it has been indicated that *γδ* T-lymphocytes play a role in antibacterial immunity and may provide a unique barrier function for mucosal microenvironments against bacterial pathogens [49]. The WC1^+^ subpopulation represents a minor portion of *γδ* T-lymphocytic population in normal MG secretions [28, 86], but they markedly increase following parturition [87]. Because of restricted localisation and expression of invariant Ag receptors, the exact contribution of these cells to MG immunity is not fully understood. Several lines of evidence have been accumulated suggesting that these cells perform specific functions in comparison to circulating *αβ* and *γδ* T-cells. Recently [88], it has been addressed that *γδ* lymphocytes exert some immunoregulatory/suppressive functions, more precisely in the WC1.1^+^ and the WC1.2^+^ cells. On the other hand, it has been reported that WC1^+^ cells are not recruited to the MG during chronic IMIs caused by* Staph. aureus* [86].

One of the main roles of B-lymphocytes is to produce Abs against invading pathogens. Unlike macrophages and PMNs, B-lymphocytes utilise their cell surface receptors to recognise specific pathogens and then internalise, process, and present Ags in the context of MHC class II molecules to T-helper cells [4]. Under certain conditions, B-lymphocyte differentiation can be directly stimulated by an Ag such as lipopolysaccharides (LPS) [49]. In contrast to T-lymphocytes, the percentages of B-lymphocytes remain fairly constant regardless of lactation stage [49, 77] or infection [86].

NK cells are large granular nonimmune lymphocytes that differentiate and mature in bone marrow, lymph nodes, spleen, and tonsils before passing to the circulation. NK cells constitute the third type of cell derived from lymphoid progenitors that also generate B- and T-lymphocytes [30]. NK cells utilise their Fc receptors to possess a cytotoxic activity critical to the IIS in the absence of MHC restriction [7]. NK cells cause lysis of target cells through a diverse repertoire of mechanisms [89], including Ab-dependent cell-mediated cytotoxicity, granule exocytosis, release of cytolytic factors, and receptor-mediated Ag-recognition. Additionally, they secrete various toxic molecules that may initiate apoptosis in altered cells [49]. Bovine NK cells, however, have not clearly shown immunoregulatory functions [88]. NK cells differ from natural killer T-cells in origin, respective effector functions, and lack of specificity for Ag-recognition. However, NK cells do not require activation to kill cells that lack self-markers of MHC class I [90]. Studies have demonstrated the capability of NK cells to kill both Gram-positive (GPB) and Gram-negative bacteria (GNB) and, therefore, they may be important in preventing IMIs [91, 92].

*(2) Distribution of Cellular Components in the Bovine MG Environment.* The differences in distribution of cellular components in MG environment between healthy and inflammatory conditions are detailed in Table 1.

The distribution of leukocytes in healthy MG is somewhat variable during healthy lactating and dry periods. The percentage of PMNs tends to increase during early and late lactation, while the percentage of lymphocytes decreases [93]. Meanwhile, the proportion of macrophages is highest (68%) in the early postpartum period and lowest (21%) in late lactation [80]. During the dry period, SCC can markedly rise. The increase at the start of involution is most likely due to an influx of cells resulting from cessation of milk removal, or due to the concentration effect by removal of the liquid phase of the secretion. SCCs in milk from uninfected glands at the beginning of the dry period are usually higher than 1 × 10^5^ cells/mL milk, but by the 7th day of the dry period this count can be as high as 2 × 10^7^ cells/mL milk [32]. PMN counts are initially high in early involutional secretions, comprising 40–80% of SCC (similar to colostrum), but are reduced again from the 2nd to 4th week of the dry period and then return to lactational values in the fully involuted udder [43, 66]. Unlike in the lactation stage [43, 66] and with exception of the 1st day of the dry period in which they exhibit higher counts [94], macrophage concentrations are relatively low during the remaining part of early involution and in colostrum, with maximal proportions (30%) peaking by the mid-dry period and remaining constant until calving [42]. Lymphocyte proportions were found to increase during involution and then decrease around parturition [66]. Lymphocyte concentrations in dry secretions are approximately 3000–6000 times that in normal milk [43], and the proportions of B- and T-lymphocytes are approximately 28% and 47%, respectively, approximating proportions in peripheral blood [31, 32].

*(3) Distribution of Cellular Components in the MG Environment of Ovines and Caprines.* The milk SCCs thresholds are higher in milk of small ruminants than in bovine milk. Recent studies have indicated an upper SCC threshold of 2.5 × 10^5^ cells/mL milk in healthy ewe's udders [95] or more, up to 6 × 10^5^ cells/mL milk [74]. Similar to bovines, the macrophages are the predominant cell type (46–84%) in milk from uninfected ewes [96, 97]. Counts of macrophages were higher in early and midlactation milk than in late lactation milk [74]. The rest of the SCs population consists of PMNs (2–28%) and lymphocytes (11–20%). Meanwhile, limited data exist on changes of leukocytes population in infected ewes' MGs. Paape et al. [98] recorded an increase of PMNs percentages to 50% at a SCC of 2 × 10^5^ cells/mL milk and to 90% at a SCC over 3 × 10^6^ cells/mL milk, representing the predominant cell type at inflammatory conditions. Likewise, an increase of PMNs and macrophages counts within IMI of ewe's udder has been reported, whereas lymphocytes decreased [99].

SCC of milk from uninfected goats is higher than those of milk of uninfected bovines and sheep. Unlike cow and sheep milk where macrophages are the predominant cell type, PMNs comprise the major cell type in goat milk from both infected and uninfected MGs [100–104]. In healthy status, PMNs, macrophages, and lymphocytes comprise 45–74%, 15–41%, and 9–20% of SCs population, respectively, while epithelial cells are present in low percentage (1–6%) [98, 103, 105, 106]. With advanced lactation, the PMNs increase, while lymphocytes and macrophages percentages are decreased [104, 107]. Manlongat et al. [102] explained this late-lactation rise-up on the presence of higher chemotactic activity in nonmastitic goats udder and concluded that this phenomenon was nonpathological and could play a physiologic regulatory role in MG involution. Unfortunately, very little data exist on the distribution of these cells during IMI. A study by Dulin et al. [100] reported an elevation of PMNs to 71–86% in infected halves, while macrophages and lymphocytes percentages are being changed to 8–18% and 5–11%, respectively.

*(4) Contribution of MG Epithelium to MG Immunity.* MECs themselves are active contributors to the innate immune and inflammatory responses of MG [108, 109]. They express a range of PR receptors (PRRs), most notably the TLRs [35, 36]. Additionally, the polymeric-Ig receptor (PIgR) expressed on the mucosal epithelium facilitates the translocation of Igs, particularly IgA, across the epithelium into the alveolar lumen [110]. Upon bacterial stimulation, MECs secrete a range of innate immune effector molecules and inflammatory mediators, which contribute to attraction and recruitment of circulating leukocytes [38, 111]. It was shown that MECs secrete IL-8, a potent neutrophil chemoattractant, in the presence of GPB and their exotoxins, LPS from GNB or IL-1*β* [51, 111, 112]. However, epithelial cells from lactating MGs may also express IL-8 [113]. MECs constitute an important source for host defence components as arachidonic acid metabolites [38, 108, 114, 115], APPs [111], LF [111, 116], *β*-defensins [117, 118], cathelicidins and calprotectin [108], and LPS binding protein [BP] (LPS-BP), which is involved in host recognition of the bacterial cell wall [17, 119]. Supporting results were obtained experimentally on bovine MECs, showing also their ability to express IL-1*β*, tumour necrosis factor-*α* (TNF-*α*), IL-6, IL-8, and growth related oncogene-*α* [GRO-*α*] mRNA during infection and immune stimulation [111, 114, 120, 121].

MG epithelium may exhibit protective and phagocytic functions via the ingestion and possible digestion of phagocytosed microbes and milk components, including fat globules and casein micelles, through the formation of pseudopodia. This effect was clearer in nonlactating glands than under lactating conditions [122]. Experimental studies showed that glutaraldehyde-killed streptococci, staphylococci, and* E. coli* were phagocytosed by milk secretory cells [115]. Moreover, many peptides, proteins, and lipids which are involved in host defence and shown to have antibacterial properties (including xanthine oxidase and sphingolipids) were found in fat globule membranes, which originate from the apical membrane of the MG epithelium [123, 124].

*(5) Recognition of Invading Mastitis Causative Bacteria by Host IIS.* The initiation of rapid and effective IIR depends mainly on recognition of the infectious agent [36, 109]. IIR of MG is initiated when PRRs on the surfaces or within host cells, primarily leukocytes and MECs, bind to particular bacterial motif molecules termed pathogen/microbial-associated molecular patterns (PAMPs/MAMPs) [109, 125, 126]. These motifs can be released during replication or degradation of a microorganism [127]. Such PRRs belong to three different families, namely, the TLR, nucleotide-binding oligomerization domain- (NOD-) like receptors (NLR) 1-2, and retinoic acid inducible gene-1- (RIG-1-) like receptors, and each of these receptors recognizes a set of bacterial motifs [17, 35, 36, 109]. Activation of these PRRs initiates a signalling transduction cascade in which nuclear factor-*κ*B plays a pivotal role in coordinating multiple signals and directing expression of effector response genes, including cytokines, as well as orchestrating both the local and the systemic immune responses [35, 120, 128–130]. In this context, it was not surprising that the expression of PRRs increases in infected bovine MGs tissues and epithelia [17, 130–135].

The TLRs represent a highly conserved family of PRRs involved in microbial detection [35]. Till now, they are the best characterized bovine PRRs and they recognize a wide range of PAMPs. Thirteen TLRs have been identified among mammals, 10 of which are known to occur in cattle [17, 35, 136]. TLRs are either expressed on the cell surface or associated with intracellular vesicles [137]. Each TLR can detect distinct PAMPs derived from microorganisms. For example, TLR pairs such as TLR1/2 and TLR2/6 can recognise lipopeptides or lipoproteins, whereas individual TLRs such as TLR2, TLR4, TLR5, and TLR9, respectively, are involved in sensing lipoteichoic acid (LTA), LPS, flagellin, and 6-base DNA motif consisting of an unmethylated CpG-dinucleotide motif (CpG-DNA) [35, 36, 109, 137–140]. Besides recognizing LPS motifs, TLR4 also can recognise bacterial-derived elastases and exoenzyme-S [141, 142]. Another important PRR found on PMNs and macrophages in the MG is CD14 [143], which can bind to LPS and induces the synthesis and release of TNF-*α* [64]. Also, the role of NOD1 and NOD2 receptors of MECS in sensing peptidoglycans (PGs) of GNB has been addressed [109, 144, 145].

*(6) Contribution of Specific Bacterial Components to the Identification by Host IIS and Induction of IRs*

*Gram-Negative Bacteria (GNB).* Cell wall LPS, or endotoxin, is central to the pathogenesis of mastitis caused by GNB. LPS is considered the most potent immunostimulant of cell wall components and is the key virulence factor eliciting clinical symptoms [36, 37]. The LPS layer of the outer membrane generally contains three regions: O-specific polysaccharide chain, polysaccharide core, and lipid A. Lipid A was found to be responsible for most of the pathogenic phenomena associated with GNB IMIs, including endotoxin shock [36]. Recognition of LPS is mediated by membrane CD14, LPS-LBP, an APP present in the bloodstream, and TLR on MECs (primarily TLR4) [35, 37, 64, 146]. As a consequence, initiation of acute IR results in an intense elevation of SCC [109, 147], activation of different leukocytes and immune-related genes [148], and subsequent production of antimicrobial defence proteins and peptides (e.g., LF, LZ, and LAP), lipid mediators (e.g., cyclooxygenase-2 and 5-lipoxygenase) [149, 150], chemokines (e.g., CXCL5, CXCL8, and RANTES) [148, 151, 152], and cytokines, especially IL-6, TNF-*α* and insulin-like growth factor-1 [35, 64, 146, 151]. Additionally, binding of soluble CD14 to LPS stimulates MECs to produce leukocytic chemoattractants such as IL-8 [112, 153]. Despite the principle role of LPS in recognizing GNB by TLRs (TLR1/2 and TLR2/6), it has been illustrated more recently [109] that PGs fragments of* E. coli*, which are known to activate the cytoplasmic NOD1 receptor, could be recognized by bovine MECs and, thus, can induce inflammatory response. Although NOD1 receptor is cytoplasmic and its activation requires that the agonist is transported into the host cell [154], it is possible that PGs fragments can reach the cytoplasm of bovine MECs following invasion by* E. coli*, as proven by some authors [155]. Moreover, the expression of membrane transporters under particular circumstances including inflammation could transport PGs fragments, as was shown for muramyl-dipeptide (MDP), a potent NOD2 agonist [144, 145].

*Gram-Positive Bacteria (GPB).* In contrast to GNB, for which LPS is the major immunostimulatory molecule, several important compounds have been identified as immune stimulators for GPB species, including cell wall lipoproteins [156], LTA, which is a cell wall component of the murein capsule [36, 119], and PGs [157] in addition to secreted exotoxins [158]. Both PG and LTA have been shown to induce immune cells, including monocytes and macrophages, to produce inflammatory cytokines and chemokines [159, 160]. PG combined with LTA induced the expression of MCP-1 and a slight increase in MCP-3 chemokine expression [148].* In vitro* studies have shown that LTA alone can induce expression of several cytokines such as IL-1*β* [161, 162], IL-6, IL-8, and TNF-*α* in MECs, although to a lesser extent than LPS [125, 161–163]. Also, LTA proved to induce strongly the secretion of the chemokines CXCL1, CXCL2, CXCL3, and CXCL8, which target mainly neutrophils [161]. The role of LTA and other PAMPS as muramyl-dipeptide in stimulating IIS is not only limited to expression of specific cytokines and chemokines, but can potentiate their subsequent effects after production. The staphylococcal LTA or muramyl-dipeptide enhances the expression of immune defence genes that are induced by IL-17 in MECs* in vitro* [162]. However, it must also be considered that the virulence of bacterial compounds such as LPS and LTA may vary somewhat depending on their bacterial origin [164]. More interestingly, LPS-BP has been shown to bind LTA of GPB cell wall [119] although primarily associated with GNB infection. The induction of the gene encoding LPS-BP was observed in all tissues of MG challenged by* Staph. aureus* [17], and increased concentration of LPS-BP has been previously reported in milk and serum after IMI with* Staph. aureus* [165].

TLR2 plays a major role in the recognition of a variety of components related to GPB including LTA and lipoproteins. LTA activates cells via the TLR2/TLR6 heterodimer [119, 134, 138, 139, 166], and with physical and functional interactions with TLR1 and TLR6 it allows discriminating the lipid portion of lipoproteins [36, 166]. Meanwhile, the roles of TLR1, TLR2, and TLR6 in the recognition of PG remain controversial, and it has been suggested that PG recognition occurs mainly intracellularly rather than from the extracellular compartments [167]. Despite the principle role of TLR1 and TLR6 heterodimers with TLR2, significant increases in the expression of TLRs that recognise viral ligands (TLR3 and TLR7) were also observed in bovine MGs challenged with* Staph. aureus* [17], and a previous study [168] has shown the role of TLR7 in recognition of GPB. Similar results were observed in human monocytes in response to both* Staph. aureus* and IL6 treatment [169]. Additionally, expression of intracellular receptors may be important in recognizing* Staph. aureus* which has the potential to invade epithelial cells [170, 171].

*(7) Other Components Contribute to Humoral Defences*

*Lactoferrin [LF].* LF, an iron-binding glycoprotein, was first isolated from bovine milk in 1939 [172]. In the MG environment, it is mainly produced by the secretory epithelium and to lesser extent by PMNs [173]. Expression of LF is inversely related to alveolar development. Little or no expression of LF occurs in lactating alveoli, and moderate to high expression occurs in the epithelia lining the ducts and cisterns, while LF expression is absent at the proximal end of the teat canal [174]. The regulation of LF expression in MG appears to be reciprocal to that of the other milk proteins [175]. Although bovine colostrum contains high levels of LF (up to 5 mg/mL), these levels drop very rapidly as lactation proceeds, so that mature bovine milk normally contains 200–485 *μ*g/mL LF or less [176, 177], depending on daily milk production and lactation stage [178]. On the other hand, LF increases markedly in dry secretions, with the maximum concentrations attained after 3-4 weeks of involution (20–30 mg/mL), nearly 100-fold greater than during lactation [179]. The antibacterial effect of LF is enhanced by increased bicarbonates and low concentrations of the LF inhibitor, citrate, present during the dry period [25, 179, 180]. The increased LF concentration during involution strongly inhibits bacterial growth, and it has been suggested to contribute to the low number of naturally occurring IMIs during this early dry period [181].

LF contributes to MG immunity, immune modulation, and transcriptional activation of various molecules via several pathways. Principally, it exerts its bacteriostatic effect by competing with bacteria for available iron [182–184] or by binding to bacterial surfaces [185, 186]. Studies have shown the ability of LF to damage the outer membrane of a broad range of GNB by interacting with the lipid A portion of LPS and performing proteins in the outer membrane (porins), altering the integrity and permeability of the cell wall [185, 187, 188] and releasing LPS, which sensitizes the cell to antibiotics [187]. The binding interactions of LF to GPB are still not fully understood, although it has been shown that LF binds to specific receptors on the cell walls of several GPBspecies associated with IMIs, including* S. uberis* [189],* S. agalactiae* [190], and* Staph. aureus* [186, 191], as well as several coagulase-negative staphylococci (CNS) (e.g.,* Staph. epidermidis*,* Staph. warneri*,* Staph. hominis*,* Staph. xylosus*,* Staph. hyicus*, and* Staph. chromogenes*), hindering their adherence to and invasion of MECs [192]. One study [193] showed that although the antagonistic effect of bovine LF on the adhesion and invasion of CNS strains to MECs is weak, it significantly decreased intracellular replication rates.

Bacteria with high iron requirements are susceptible to the bacteriostatic activities of LF. Among mastitis-causing bacteria,* E. coli* are the most susceptible followed by* Staph. aureus*, but streptococci are more resistant [194]. For* E. coli*, it appears that Igs are not required for LF to exert a potent bacteriostatic effect [195].* S. uberis* was found to resist the antimicrobial effect of LF compared to* Staph. aureus* and* E. coli*, although* S. uberis* challenged MG shows increased mRNA expression of LF-related gene [196] and stimulated the production of LF more than the other two organisms [197]. In this context, some studies showed that bovine LF can enhance adhesion of* S. uberis* to host cells and increase invasiveness, suggesting that* S. uberis* has evolved to take advantage of the presence of LF [198, 199]. On the other hand, bovine LF has also been shown to inhibit many pathogenic bacteria, including* Listeria monocytogenes* [200] and enterotoxigenic* E. coli* [200, 201], and to increase the antibacterial effect of antibiotics synergistically against antibiotic-resistant GPB [186].

As a major component of the specific granules of PMNs, LF additionally contributes to both hydrogen peroxide-dependent and hydrogen peroxide-independent bacterial killing [202] and promotes the adhesion and aggregation of PMNs to the endothelial surface [203]. Another aspect of LF's antibacterial activity is based on activation of the CS via the alternative pathway [204]. LF may also be important in Ag-processing by cells of the reticuloendothelial system and in Ab production [205]. Additionally, LF increases NK cells activities [184] and amplifies the inflammatory response and stimulates the phagocytic and cytotoxic properties of macrophages against invading pathogens [203, 205] such as* Staph. aureus* [204] but still as a potent inhibitor of granulocyte-monocyte colony-stimulating factor [205, 206].

During mastitis, LF levels in lacteal secretions may increase 30-fold, corresponding to the severity of infection [111, 149, 176, 197, 207] and depending on the causative agent, as evidence has accumulated suggesting that different pathogens induce different LF-mediated responses from MECs [197]. The dramatic increase in LF concentrations in milk during acute mastitis is consistent with the role of LF as an acute phase response (APR) protein in the MG, in accordance with the presence of APR elements in the LF gene promoter region [182]. In experimentally induced* E. coli* mastitis, the mean concentration of bovine LF was 2 mg/mL [208], whereas in CNS mastitis it was <0.2 mg/mL [207]. The expression of LF by MECs* in vitro* has been shown to be greater upon exposure to* S. uberis* isolated from acute mastitis compared to* S. uberis* isolated from chronic mastitis [209]. Based on the strong association between LF concentrations and mastitis occurrence, combined with the antibacterial properties of LF, it has been suggested that bovine milk LF plays an important role in defence against* E. coli* if concentrations exceed 200 *μ*g/mL milk [185, 188, 210], while it has little effect against other major pathogens such as* Staph. aureus* and* S. agalactiae* [210, 211].

*Transferrin [TF].* TF is another iron-BP in the milk of dairy ruminants, although it is present at low concentrations [25]. It was first isolated in 1960 from both human and bovine milk [212]. The concentration of TF ranges from 1.07 mg/mL in colostrum to 0.02–0.04 mg/mL in milk of third week postpartum compared to 4-5 mg/mL in serum [213, 214]. In contrast to rodents, pigs, and rabbits, which synthesise TF in the MGs at higher concentrations, TF in the milk of dairy ruminants is not synthesised in the udder [116] and instead comes from blood serum, from transcytosis in the normal gland, and through exudation of plasma during mastitis [215]. Like LF, TF can damage the cell membranes of GNB with the release of LPS, thereby altering outer membrane permeability [188]. During experimental* E. coli* IMIs in dairy cows, TF concentrations were found to rise even before LF elevation, reaching 1 mg/mL in milk and paralleling the concentrations of serum albumin [180].

*Lysozymes (LZs).* LZ (N-acetylmuramyl hydrolase) is one of the components of antibacterial system in milk [4, 216, 217]. LZ has inhibitory or lytic activity mainly against GPB and to lesser extent against GNB by cleaving the *β* 1,4-glycosidic bond between N-acetylmuramic acid and N-acetyl-D-glucosamine residues in PG [217], thereby disrupting the cell wall [4, 177]. However, milk LZ alone is not a significant component of the BMG defence, and only a few mastitis-causing bacteria are killed by LZ. Nonetheless, LZ can synergize with Abs, complement, and LF [4, 25]. For example, the binding of cationic LF to the LTA of GPB renders staphylococci more susceptible to LZ [4, 218]. In healthy conditions, LZ concentration of milk shows wide variation among species and is influenced by several factors such as the period of lactation, health, age, and the parity of animals [217, 219]. After parturition, the LZ concentration shows successive increase, reaching the peak (0.72 mg/L milk) at the 7th day, and then begins to decrease after the 2nd week postpartum [220]. Nevertheless, bovine and buffalo milk contain averages of only 0.0004 and 0.000152 g LZ/L milk, respectively, compared to 10 mg LZ/100 mL in human milk [221]. A substantial rise (10–50-fold) of lysosomal activity of milk has been recorded during mastitis among different dairy species [149, 217, 222, 223]. However, buffalo may exhibit thousandfold greater LZ activity and moderately raised SCCs in milk without showing signs of mastitis [224]. LZ in milk may be derived from blood or locally synthesized [8], and during IMI leucocytes appear to be the source of LZ [173].

*Lactoperoxidase and Myeloperoxidase Systems.* Next to xanthine oxidase, lactoperoxidase is the most abundant enzyme in milk, constituting 0.5% of the total whey proteins (30 mg/L^−1^) [225, 226], and nearly similar concentration is present in colostrum [226, 227]. As for many other indigenous enzymes, the level of lactoperoxidase in milk increases with mastitis [228]. Locally synthesised lactoperoxidase, in the presence of thiocyanate of hepatic origin and hydrogen peroxide of either bacterial or endogenous origins, can exert antibacterial properties against both GPB and GNB via the generation of activated oxygen products like hypothiocyanate, a reactive metabolite formed from the oxidation of thiocyanate that promotes bactericidal activity of phagocytes [5, 177]. A close relationship between lactoperoxidase activity and SCC in goat milk has been reported [229]. It has been hypothesized that lactoperoxidase may have a synergistic antimicrobial function with lingual antimicrobial peptide (LAP), one of the host defence peptides, in MGs of dairy cows [150].

Myeloperoxidase is a lysosomal enzyme similar to lactoperoxidase [225]. It is mainly located in the primary granules of neutrophils [230], and together with peroxide and halide it has an important role in the oxygen-dependent antimicrobial system of neutrophils and thus in defence against microorganisms [231, 232]. It catalyses the same peroxidase reaction as lactoperoxidase and additionally catalyses the oxidation of chloride, the product of which provides the bactericidal activity of this system [4].* In vitro*, this system has been shown to be potent against major common udder pathogens such as* Staph. aureus*,* S. agalactiae*,* S. dysgalactiae*,* S. uberis*, and* E. coli* [230]. Unfortunately, the antibacterial properties attributed to this system are only relevant during the dry period, whereas they were found to be completely inhibited with lactation, mainly due to milk proteins [230]. Additionally, the levels of thiocyanate in udder are dependent on the specific dietary composition, and the low oxygen tension of the MG can inhibit the production of hydrogen peroxide, thus limiting the effectiveness of this antimicrobial system against different pathogens incriminated in mastitis [4].

*Complement System (CS).* Complement is a collection of proteins that are produced in plasma mainly by liver as well as tissue macrophages and monocytes and for C3 a local synthesis in the MG was suggested [233]. In support of the assumption of a local synthesis, experimental* Staph. aureus* and* E. coli* IMIs induced an increase of C3 mRNA-expression in MECs [134]. Complement components elicit their biological activities through complement receptors located on a variety of cells [7, 134, 233]. The CS is central to IIS because it is intimately involved in initiation and control of inflammation, opsonisation of bacterial surfaces, attraction and recruitment of phagocytes (chemoattractants) (e.g., C3a and C5a cleavage fragments), recognition and ingestion of microorganisms by phagocytes (e.g., C3 and C4), and the killing of microorganisms, either directly or through cooperation with phagocytic cells [53, 134, 233–235]. Nevertheless, it was also gradually appreciated that different proteins of the CS can influence the MGIR and constitute an important bridge between IIS and AIS [53, 235, 236].

The lowest concentrations of complement are observed in the milk of healthy MGs during lactation, and higher levels are observed during late lactation period, in colostrum, and in mammary secretions obtained during involution, presumably due to the mobilisation of complement components by transudation from blood [237–240]. The alternative pathway (AP) was found to be the sole complement pathway operating under these healthy conditions, while the classical pathway (CP) is not functional due to lack or lowered presence of C1q component compared to blood [53, 233, 241]. The AP operates with two consequences that are greatly involved in recruitment and activation of phagocytes, mainly PMNs: (1) deposition of opsonic C3b and C3bi on bacteria and (2) generation of the proinflammatory fragment C5a [75, 234, 241, 242]. However, the milk from noninflamed MG is generally devoid of significant haemolytic and bactericidal complement-mediated activities, especially during the midlactation period [240, 241, 243, 244], due to strong anticomplement activity of milk on complement mediated hemolysis and the absence of the C1q component required for activation of the CP [5, 177, 244], except for some healthy periods of exerting elevated complement concentrations, where these activities exist in a weak but significant manner [237, 238, 240]. Nevertheless, this inhibitory activity does not involve C3b/C3bi deposition on bacteria or the generation of C5a by the AP [233]. Unfortunately, the lack of haemolytic activity in bovine normal milk in the absence of inflammation adversely affects a very important function of the CS, opsonisation of bacteria by CS components, mainly C3 [243]. However, it has been shown a noteworthy deposition of C3 complement fragments from neat milk of non inflamed MG on some particular udder bacteria, as mastitis-causing* Staph. aureus* [53], and* S. agalactiae* even in mid-lactating period [241] by the activation of the AP. In addition, an enhanced chemiluminescence response of PMNs against invading pathogens was noticed [53, 245]. On the other hand, the production of extracellular fibrinogen-BP by* Staph. aureus* was found to inhibit complement activation by blocking C3 deposition on the bacterial surface [246].

In contrast, the highest concentrations of complement are observed in mastitic milk, presumably due to the mobilisation of complement components by transudation from blood [233, 238, 239]. Relative to the increase in complement concentrations during IMI after recruiting plasma components, both bactericidal and haemolytic activities of CS are increased in inflamed MG, and the intensities of these activities correlate with intensity of the IR [233, 247, 248]. GNB (e.g.,* E. coli*) are sensitive to complement lytic action, while some GPB (e.g.,* Staph. aureus*) are resistant, although all bacteria show susceptibility to the opsonizing action of C3b and C3bi fragments after activation of the AP [53, 233, 241, 247, 248].

*Cytokines, Chemokines, and Growth Factors.* Cytokines are water-soluble regulatory peptides produced during inflammatory processes. Most cytokines have more than one function and often have redundant effects with other cytokines [249]. Because of the high affinity of their receptors, cytokines are highly potent and can elicit biological responses even at femtomolar to nanomolar concentrations [250]. Numerous cytokines (e.g., TNF-*α*, IFN-*γ*, GM-CSF, IL-8, and IL-12) have been detected in normal udders [251, 252], but during IMI a complex upregulation of specific cytokines occurs depending on several factors. Cytokines act at both local and systemic levels during onset, progression, and resolution of inflammation [253, 254]. They provide relatively short-range communications between cellular immune components, thus linking the innate and adaptive immune branches [255], and this short communication range is important to limit their effects to the appropriate cells. Although cytokines play an essential role in the host response to infection, they can also have deleterious effects. Thus, there is a fine balance between the positive and negative effects of cytokines on the host that is dictated by the duration, amount, and location of their expression [256]. A more detailed explanation of the roles of specific cytokines, chemokines, and growth factors in MG during IMIs is illustrated in Table 2.

Due to their important contributions to the inflammatory process, several studies have illustrated cytokines benefits in immunotherapy of mastitis via enhancing MG immunity (e.g., interferons, mainly IFN-*γ*, IL-2) [257–261], their contributions to control or prevention/immunisation against mastitis pathogens especially* E. coli* or* Staph. aureus* (e.g., G-GSF, GM-CSF, IL-2, and IFN-*γ*) [262–264], and their potentiating effects on response to treatment with antibiotics (e.g., IL-1, IL-2, and IFN-*γ*) [262, 265–269]. The efficacy of recombinant cytokines (e.g., recombinant bovine IL-2 [rBOIL-2]) in accelerating the involution of MG during dry period, and thus reducing the time in which the MG is particularly susceptible to infection, has been addressed [270, 271]. Intramammary infusion of IL-2 elicits a considerable increase in SCC, which is dominated by macrophages and plasma cells producing IgG1, IgG2, IgA, and IgM. On the contrary, the immunotherapeutic properties of rBOIL-1 are masked by the domination of proinflammatory nature of IL-1 [251, 271, 272].

Chemokines are important molecules involved in migration and recruiting leukocytes into MG during IMI, besides being involved in several immunoregulatory and inflammatory processes [39, 51, 151, 161]. According to arrangement of conserved N-terminal cysteine motifs, chemokines are grouped into 4 families: C, CC, CXC (subdivided into ELR^+^ and ELR^−^), and CX3C [151]. Members that contain the motif (ELR^+^) are potent chemoattractants for neutrophils and promoters of angiogenesis, whereas those that do not contain the motif (ELR^−^) are potent chemoattractants for mononuclear cells [151, 161]. Representatives of the ELR^+^ CXC chemokines are structurally similar, including IL-8/CXCL8 and ENA-78/CXCL5 [273]. Chemokines target neutrophils by interacting with one (e.g., CXCL1, CXCL2, and CXCL3) or two (e.g., CXCL8) receptors, CXCR1 and CXCR2, which are expressed by neutrophils of several species including cattle [274]. Several molecules which mediate leukocytic trafficking are expressed in the MG tissues and MECs in response to LTA from GPB (e.g., CXCL1, CXCL2, CXCL3, and CXCL8) or LPS from GNB (e.g., RANTES, CXCL5, CXLX8, MCP-1, MCP-2, and MCP-3) and can be also detected in milk [39, 51, 127, 135, 151, 152, 161, 165, 275]. The remarkable induction of chemokine gene expression by the epithelial cell lends strong support to its role in stimulating migration of leukocytes into the MG [39, 63].

*Host Defence Peptides (HDPs).* Host defence peptides (HDPs) are a large family of innate immune effector molecules. They are predominantly synthesised in PMNs and epithelial cells [56–58, 132, 276] and have been shown to be important in the resolution of local infection through both antimicrobial and immune-regulatory properties [58]. Defensins are an important family of HDPs in cattle owing to variable bactericidal properties [57, 276] and are considered as effector arm of IIS as well as representing a putative link between IIS and AIS [58, 117, 132, 277]. Several *β*-defensins, including LAP, tracheal antimicrobial peptide (TAP), and bovine neutrophil *β*-defensins 1, 4, and 5 (DEFB1, DEFB4, and DEFB5), are expressed in MG tissues in both a constitutive and an inducible manner, or even excreted in milk, in response to bacterial challenge [17, 57, 117, 118, 131, 132, 150, 278]. Also, an increase in LAP mRNA expression in the bovine alveolar tissue at 192 h after milking upon involution has been declared [40]. A broad spectrum of antimicrobial activities has been demonstrated for several bovine *β*-defensins, in particular against several species that cause mastitis as* Staph. aureus*,* E. coli*,* Kl. pneumoniae*, and* Ps. aeruginosa* [57, 118, 279].

#### 2.2.2. AIS and Its Related Components

The specific or adaptive immune system [AIS] recognises specific determinants of a pathogen mainly via Abs molecules, macrophages, and several lymphoid populations, which subsequently facilitate selective elimination [7, 27]. Because of the memory function of certain lymphocytes, specific IRs can be augmented by repeated exposure to a pathogen [7]. Immunoglobulins (Igs) are the most important specific soluble humoral factors in adaptive immune defence, linking various parts of the cellular and humoral immune system, and they constitute the main component of the AIS present in colostrum and milk [33, 280]. They are able to prevent adhesion of microbes to tissues, inhibit bacterial metabolism, agglutinate bacteria, augment opsonisation and phagocytosis of bacteria, kill bacteria through activation of complement-mediated bacteriolytic reactions, and neutralize toxins and viruses [281, 282]. Igs account for up to 70–80% of the total protein content in colostrum (20–150 g/L) to confer passive immunity to newborns, whereas in milk they account for only 1-2% of total protein (0.5–1 g/L) [31, 226, 227, 247]. However, Ig concentrations in the BMG vary during the lactation cycle, and an increase occurs at the end of lactation [283]. The Ig content of both milk and colostrum increases during inflammatory conditions [8].

Igs in milk may be blood-derived or may be produced* in situ* by Ag-activated plasma cells, which traffic to the udder from the blood [77, 284] mediated by chemokines produced locally during IMI [285]. The MG plays an active role in regulating the levels of different Igs present in colostrum and milk, although the mammary epithelium itself does not synthesise Igs. The majority of Igs are transported into mammary secretions via specialised receptors (selective receptor-mediated intracellular route) [33]. There are four different classes of Igs that play dominant roles in MG defence against bacterial pathogens: IgG1, IgG2, IgM, and IgA (Table 3). Functionally, IgG1, IgG2, and IgM act as opsonins and facilitate phagocytosis by PMNs and macrophages [49, 247], while IgA is thought to play roles in toxin neutralisation and bacterial agglutination, thereby hindering bacterial spread and colonisation [247, 284]. Bovine colostrum contains IgG1, IgA, and IgM in concentrations exceeding those of blood. The colostrum/blood ratios for IgG1, IgA, and IgM are approximately 4 : 1, 13 : 1, and 2 : 1, respectively [286]. The most abundant Ig class in bovine milk and colostrum is IgG1 [287–289], while IgG2 increases substantially during inflammatory states [247]. In contrast, IgA and IgM are present at much lower concentrations in healthy BMGs [286, 290].

## 3. Coordination of MG Innate and Adaptive Immune Arms during IMI

As mentioned, both innate and adaptive IRs are coordinating and operating together in very complicated pathways to provide the optimal defence against infections. PR and Ag presentation by innate immune components initiates a proinflammatory response with quantitative and qualitative changes of different immune components in a complex manner. Different cytokines and chemokines appear to play essential roles in this process by acting through their variable immunoregulatory roles, thus coordinating MGIR.

Once bacteria contact leukocytes in the milk or the lining MG epithelium accompanied by exerting various virulence mechanisms and liberating toxins, irritation or even damage to MG epithelium and, thereby, activation of the IIS occur through the transcriptional activation of key response genes [126]. Inflammatory products from damaged epithelium induce locally located leukocytes and healthy MG epithelium to release several chemoattractants for the migration and recruitment of both bone marrow and circulating immune cells into the MG environment, mainly neutrophils [39, 63, 151, 255, 291, 292]. Proinflammatory cytokines (IL-1*β*, IL-6, and IL-17) as well as IL-8 and TNF-*α* are the main effectors to initiate the inflammatory responses at both local and systemic levels [121, 162, 291, 293, 294]. They act in collaboration with TGF-*α*, GM-CSF, and several chemotactic factors (e.g., C3a and C5a complement fragments, leukotriene B4, PAF, eicosanoids [as Prostaglandin-F2*α*], oxygen radicals, and APPs) to potently trigger circulation-into-MG migration of neutrophils via induction of vascular endothelial adhesion molecules expression (mainly for E- and P-selectins), thereby promoting neutrophil transendothelial migration to the infected foci [291, 295, 296]. As a consequence, enhanced expression and adhesiveness of another neutrophil adhesion molecule, Mac-1 (known also as CD11b/CD18), occur, which allows neutrophils to bind tightly to activated endothelium in collaboration with another endothelial adhesion molecule, ICAM-1 [27]. This adhesive interaction allows neutrophils to migrate along the endothelial surface and into MG tissues up a concentration gradient of chemoattractants; one of the most potent with long-lasting effect is IL-8 [75, 256, 291, 292, 297]. It is thus clear that the migration of immune cells to MG is not a random process and a collaboration of several molecules, chemoattractants, selectins, and integrins is greatly needed to regulate chemotaxis. IL-17 has been suggested to enhance leukocytic recruitment into MG via regulating IL-8 expression and enhancing expression of several chemokines targeting not only neutrophils (CXCL3 and CXCL8) but also mononuclear leucocytes (CCL2, CCL20) [121, 162, 294].

Leukocytes that freshly migrated express greater numbers of cell surface receptors for Igs and complement and are more phagocytic than their counterparts in blood [49]. Stimulation of microbicidal activities of various leukocytes located inside infected tissues is mainly regulated by certain proinflammatory cytokines (Table 2). The activation status and enhancing functions of neutrophils are stimulated mainly by IL-1, IL-8, IFN-*γ*, TNF-*α*, and G-CSF; macrophages by IL-12, M-CSF, and GM-CSF; and NK cells by IL-2 and IL-12. Meanwhile, B-lymphocyte differentiation is driven mainly by IL-2 and IL-6 [27, 256, 298–305].

Systematically, several physiologic responses occur as a result of IMI: (1) generation of febrile response [293, 296, 301, 306, 307], (2) alterations in metabolism and gene regulation in the liver, resulting in elevation of APPs levels as well as serum cortisol levels [308], and (3) changes in vascular permeability, tone, and activation [257, 293, 296, 309]. Some cytokines such as TNF-*α*, IL-1*β*, and IL-6 are responsible for generation of febrile response, and the latter one specifically contributes to the great extent for regulation of the APR through the synthesis of APP [310]. IL-17 greatly synergizes to generation of inflammatory reactions via enhancing production of IL-6 [121, 162], IL-8, and Gro*α* [121] and the expression of inflammatory cytokines TNF-*α* and IL-1*β* [162] (Table 3). Likewise, TGF-*α* has been shown to have a potential role in mediating IIR and promoting inflammation by upregulating the production of prostaglandins and synergistically enhancing the effects of IL-1*β* and TNF-*α* [311–313]. Additionally, TGF-*α* has the ability to directly stimulate IL-8 [313] and to induce expression of antimicrobial peptides [314].

Ags from invading mastitis-causing bacteria are processed mainly within macrophages and B-lymphocytes and appear on the membranes in association with MHC class I or II; thus they can be recognised by different lymphocytes [27, 45, 67–69]. IFN-*γ* greatly contributes to upregulating of the MHC-I expression and MHC-II Ag presentation, thus increasing cytotoxic T-cell recognition for foreign peptides, and inducing CD4+ T-cell activation [256, 303]. Upon recognition of Ag-MHC class II on B-lymphocytes or macrophages, CD4+ cells are activated and produce cytokines that have roles in the activation and polarisation of B- and T-lymphocytes, macrophages, and various other cells that participate in the IR [4, 7, 27, 81]. Depending on the repertoire of cytokines produced, the T-helper cell response can facilitate either a cell-mediated (Th1 type) or a humoral (Th2 type) IR [315]. IL-2 and IFN-*γ* are the major cytokines secreted by Th1 cells, and they stimulate cellular responses against intracellular pathogens. In contrast, IL-4, IL-5, and IL-10 are secreted by Th2 lymphocytes; these cytokines promote humoral immunity and regulate both macrophage functions and the activity of cytokine production [27, 316]. On the other hand, inflammatory-inducer IFN-*γ* and regulatory IL-4 are the main cytokines produced by CD8+ cytotoxic and CD8+ suppressor T-cells, respectively. Inflammatory cytokines produced by T-cells in turn induce the proliferation and differentiation of the B-lymphocytes into either Ab-producing plasma cells or memory cells [27, 49], and some of them are responsible for increasing Fc receptors for IgG2 [81]. Synergistically, activated macrophages release chemotactic signals for neutrophils, thereby amplifying the inflammatory response [67]. Macrophages secrete prostaglandins and leukotrienes that augment local inflammatory processes [75, 317] as well as specific cytokines that are known to regulate T-cell differentiation, mainly IL-12 [318].

Regulation of polarising T-helper subsets into either Th1 or Th2 is the main axis on which some regulatory cytokines (IL-4 and IL-12) work [319]. IL-12 is produced in response to bacteria and bacterial products [318]. IL-12 contributes to the IR by favouring the polarising CD4+ T-cells towards Th1 responses and enhancing the generation of cytotoxic-IFN-*γ* producing CD8+ cells and also acts as a growth factor for NK cells and an inducer of their cytotoxic activities [254, 318, 320]. Thus, it contributes to the production of IFN-*γ* from lymphocytes as well as NK cells [254, 318]. In contrast to IL-12, IL-4 favours the development of Th2 subsets and exerts a clear inhibitory effect on IFN-*γ* production [321]. Compared to the anti-inflammatory IL-10 cytokine, the inhibitory effect of IL-4 on monokine synthesis is lesser [322]. Based on the effects of IL-4 and IL-12 on polarisation of T-cell subsets, the early preference expressed in the IR is greatly dependent on the balance between IL-12 and IL-4 [318].

Resolution of the IMI is mediated by upregulation of several inflammatory-antagonist cytokines, including IL-10, and TGF-*β*, and in corporation to anti-inflammatory effects elicited by IL-6 and IL-4. IL-10 is the most potent contributor to this process as it downregulates both the generation of all subtypes of T-helper cells [323] and the production of proinflammatory cytokines, chemokines, and eicosanoids by monocytes, macrophages, and neutrophils [85, 253, 291, 324]. IL-10 potently inhibits the ability of macrophages to stimulate Th1 cells to produce cytokines, principally IFN-*γ* [316], and has an inhibitory effect on LPS-induced production of IL-1, IL-6, and TNF-*α* by macrophage cell lines [322]. In cooperation with IL-6, IL-10 also upregulates IL-1 receptor antagonist and soluble TNF receptors, impairing the ability of the proinflammatory cytokines IL-1 and TNF-*α*, respectively, to exert their effects [256]. In contrast, IL-10 does not inhibit cytokine production by B-lymphocytes nor does it affect the ability of different phagocytes to stimulate cytokine production by Th2 cells [316]. Like IL-10, the major role of TGF-*β* is to suppress the IRs, although some proinflammatory properties have been reported [325, 326]. The anti-inflammatory role of TGF-*β* is exerted through its ability to (1) inhibit macrophage production of chemokines, proinflammatory cytokines, nitric oxide, and ROS; (2) limit IFN-*γ* production; (3) increase expression of the IL-1 receptor antagonist; and (4) enhance macrophage clearance of bacteria and cellular debris [325, 326]. The repair of damaged MG epithelium is mainly mediated by TGF-*α*, which promotes epithelial proliferation and tissue remodelling [327]. TGF-*β*, on the other hand, promotes extracellular matrix deposition, fibrosis, and scarring [328]. Thus, restoring healthy structure/homeostasis and scar formation is controlled by the balance between the two TGF types. During the whole process, altered cells are mainly removed by macrophages and cytotoxic T-cells, which recognise and eliminate altered self-cells via Ag presentation, with the help of *γδ* T-cells and NK cells, which mediate cytotoxicity with variable involvement of MHC molecules [27, 83, 84].

## 4. MGIR towards Certain Common Mastitis-Causative Bacteria

In addition to investigating the pathogen virulence mechanisms and the resulting histopathological changes, study of the immunological profile of the MG against a particular pathogen will help provide a better understanding of the nature, rate of development, and severity of mastitis caused by such pathogen and is considered a prerequisite to the development of novel and effective diagnostics and therapeutics. The sensitivity and responsiveness of the MG in terms of specific immune factors varies greatly against different bacteria [37, 131, 134, 165, 196, 275, 329–331] and their associated toxins [125, 148, 332, 333]. Thus, the high sensitivity of the MG to some mastitis pathogens results in a robust IR, invoking an acute response to infection and likely predisposing to rapid elimination of the invading bacterium with proper host immunity and animal management. In contrast, the failure to eliminate certain bacteria as* Staph. aureus* and some CNS may result in subclinical or chronic IMIs as a result of poor responsiveness of MG immunity. In attempt to understand the pathogenesis of IMIs caused by different bacterial species, several studies have assessed the mammary IRs towards particular mastitis pathogens, as shown in Table 4.

Unfortunately, most studies regarding mammary IRs towards particular pathogens in bovines have focused on* Staph. aureus* and* E. coli*, being of the most commonly mastitis-incriminated bacteria. Most bacterial species causing coliform mastitis elicit a marked acute inflammatory response in comparison to* Staph. aureus*, mainly due to the presence of LPS. However, the IIR varies among different mastitis-causative species. A strong TNF-*α* response to LPS was found to be central to the earliest initiation of MGIRs and in the development of pyrexia associated with coliform mastitis, endotoxic shock in per acute form [127, 334, 335], leukopenia in peripheral blood, and concurrent increases in milk leukocytes [62, 336, 337]. The powerful chemotaxis and recruitment of leukocytes, mainly PMNs, and robust production of a wide variety of cytokines reflect the MG's sensitivity to and response against* E. coli* compared to* Staph. aureus* [114, 127, 131, 165, 239, 307, 311, 329, 338, 339]. When similar concentrations of heat-inactivated* E. coli* and* Staph. aureus* bacteria were used to stimulate isolated MECs, expression of TNF-*α*, IL-1*β*, IL-6, and IL-8 was greater in cells stimulated by* E. coli* [134]. Experimental studies conducted on ovines revealed similar results regarding MGIR towards* E. coli*, and increases in leukocyte recruitment (mainly PMNs) and proinflammatory cytokine levels (including IL-1*β*, IL-8, and TNF-*α* [255, 340]) have been reported in response to either* E. coli* or its endotoxin. Occasional increases in GM-CSF and IFN-*γ* have also been shown [255, 340]. These data explain why* E. coli* IMIs follow acute form and why these IMIs may resolve spontaneously within a short period as declared in previous studies [341, 342].

Depending on the levels of chemoattractants and proinflammatory, inflammatory, and regulatory cytokines, the IIR is also robust towards* Kl. pneumoniae* [275] and* Ps. aeruginosa* [37], reflecting the strong MGIR towards these bacteria. Against* S. marcescens*, however, the MGIR is comparatively modest [337, 343]. The number of bacteria isolated from MGs of* S. marcescens*-infected cows as well as SCCs dropped precipitously 24 h and 48 h following infection (PI), respectively, which could reveal elimination of bacterium by MG immune system [343]. Though several studies reported strong systemic responses and clinical signs in animals infected with several species of GNB [343–345], the accurate investigations focused on the IIR towards GNB other than* E. coli *are considered rare and mostly experimental. Further* in vivo* and* in vitro* studies are required. Although* Ps. aeruginosa* elicits a strong MGIR, studies on* Ps. aeruginosa* infection in humans have revealed that secretion of exotoxin A, exoenzyme S, and elastase by such bacterium inhibits monocyte and neutrophil chemotaxis and respiratory burst, thus altering the IR [346, 347].

Unlike the case with* E. coli*, MGIR against* Staph. aureus* was found to be insufficient to eliminate the bacterium, allowing persistence of infection and eventually leading to subclinical or chronic patterns of IMI. Comparative studies [131, 135] showed that* E. coli* but not* Staph. aureus* IMI induced strongly IL-8 and TNF-*α* gene expression in the MG tissue as well as strong activation of NF-*κ*B in MECs [135] and triggered a rapid early expression of *β*-defensin, TLR2, and TLR4 in the inoculated MG and lymph nodes [131], while impaired proinflammatory activation was paralleled by a complete lack of NF-*κ*B activation in MECs challenged by* Staph. aureus* or LTA [135], and only expression of *β*-defensin occurred later than 48 h in inoculated quarters with* Staph. aureus* [131]. In a contradictory study [17], although all 10 TLRs' and NOD 1-2 expression was upregulated in MG tissues challenged with* Staph. aureus*, with TLR8 having the least expression in comparison to the other PRRs, immunohistochemistry analysis of tissues from both* Staph. aureus* challenged and control animals reported low levels of immune cells. This variability in the expression of PRRs could be attributed to different strains, but in all conditions how the IR of MG towards* Staph. aureus* is being translated remains as a crucial point. In the last study [17], expression of proinflammatory cytokines (IL6, IL17A, and IL8) and anti-inflammatory cytokine (IL10) was induced in infected MG tissues with* Staph. aureus*. Meanwhile, the production of these cytokines varied among studies (Table 4), which reveal the complexity of MGIR towards* Staph. aureus* and illustrate that MGIR could be modulated due to pathogen factors suppressing the production of these cytokines. Reduced expression and induction of some inflammatory cytokines, including TNF-*α* by LTA, the principle immune-stimulator of Gram-positive cell wall [17, 125, 126, 163], impaired activation of NF-*κ*B [135] and reduced expression and production of chemokines (IL-8 and RANTES) [134, 165], involved in recruiting leukocytes, which may reflect why the SCCs are not elevated in MGs challenged by* Staph. aureus* as SCCs from MGs challenged by* E. coli* did. It has been hypothesized that decreased expression of immune-modulator *α*-1 acid glycoprotein in the alveolar region of MG experimentally challenged with* Staph. aureus* may inhibit the early recruitment of neutrophils to the MG and could be a result of modulation of the host's IR by the pathogen in order to enhance survival [17]. Also, since it has been suggested that TGF-*β* was found to block the TLR signalling [348], the expression of TGF-*β* in IMI caused by* Staph. aureus* was suggested to be a reason of impaired IR towards this pathogen [135]. Additionally, various studies have shown that staphylococcal enterotoxins (SEA, SEB, SEC, and toxic shock syndrome toxin-1) act as super Ags by activating specific types of T-lymphocytes (mainly CD8+ suppressors) and stimulating release of specific cytokines [332, 333, 349]. The presence of high numbers of suppressor CD8+ T-cells compared to CD4+ T-cells significantly suppresses lymphocyte IRs and recruitment [78, 86]; and in addition to unstable expression and release of inflammatory inducers (IL-1*β*, IL-8, and TNF-*α*) [17, 86, 114, 131, 134, 165, 239, 329], compromised expression and release of inflammatory cytokines (depressed IL-2 and C5a levels) [17, 86, 114, 239, 350] and unstable release of anti-inflammatory IL-10 [114] could greatly reflect and provide explanation for the suppressive nature of mastitis-causative* Staph. aureus* and why IMIs caused by such bacterium do not usually undergo resolution and follow subclinical or chronic patterns with persistence of the pathogen.

CNS are known to cause IMI mainly of subclinical nature. In addition to causing a marked increase in SCC, CNS can persist similar to* Staph. aureus* and cause a similar type of histopathological MG damage [351–353]. Unfortunately, few studies have investigated the bovine MGIR against CNS, and the majority were conducted in ovines or investigated only few aspects of MGIRs. In both bovines [354] and ovines [355, 356], the IMIs caused by* Staph. epidermidis* and* Staph. simulans* were associated with a decline in leukocyte counts for a short period after initiation of the inflammatory process [355] and the absence of a marked systemic cytokine response [354]. However, some proinflammatory cytokines, including IL-1*β*, IL-8, and TNF-*α*, were elevated in milk [354–356]. These observations likely reflect the unsuccessful combat of MG against the invading bacterium and that the sensitivity or responsiveness of MG to inflammatory signals decreased as infection progressed. In experimentally induced ovine IMI by* Staph. epidermidis*, counts of leukocyte subsets (including CD4, CD8, WC1, and MHCII) temporarily decreased and then subsequently increased, while the expression of some adhesion molecules (CD11b and CD18) on PMNs decreased after 24 h [355]. An experimental study [357] in bovines revealed a mild host IR against* Staph. chromogenes* as measured by systemic signs, SCC, milk yield, bacterial counts, and some inflammatory indicators (including enzymatic activity and APP levels), but cellular and other soluble factors of MG immunity have not been studied. In addition,* Staph. warneri*,* Staph. simulans*,* Staph. chromogenes*, and* Staph. xylosus* have been shown to cause cellular responses in both ovines [358, 359] and caprine [360] udders, as indicated by increased SCC and leukocyte counts in milk and severe infiltration of MG tissues with mononuclear cells and neutrophils on histopathological investigations. Further studies are required regarding the MGIR towards several species of CNS.

Unfortunately, few studies have focused on MGIRs against streptococci, despite their substantial contribution to mastitis. To our knowledge, only one study [361] has evaluated the MGIR towards* S. dysgalactiae *subsp.* dysgalactiae*, and few studies were conducted on* S. uberis* [196, 209, 336, 337, 362–364]. Although not completely comprehensive, MGIR towards* S. dysgalactiae* subsp.* dysgalactiae* in one study was represented by increased expression of TLR4 plus release of various cytokines (IL-1*β* and TNF-*α*).

Most experimental challenge studies showed that MGIR against* S. uberis* was not sufficient to allow successful elimination of the bacterium, although increased expression and production of several inflammatory mediators and antimicrobial components as IL-1*β*, IL-8, IL-10, IL-12, IFN-*γ*, TNF-*α*, sCD14, LPS-BP, C5a, and LF have been declared during IMIs caused by* S. uberis*. In* S. uberis*-experimentally infected cows, both numbers of bacteria in milk and SCCs remain highly elevated for long time PI, compared to* S. marcescens* infected cows [343]. Neither the influx of PMNs into MG infected with* S. uberis* [336, 337, 365] nor intracellular engulfment by macrophages [70, 366], have resulted in effective reduction in the number of bacteria, and in contrast intracellular replication of* S. uberis* inside macrophages increased. Additionally, it has been accumulated that MGIR towards* S. uberis* is very complex, and different strains of* S. uberis* can elicit different IRS. Some studies showed that strain-specific pathogenicity greatly modulates the IR, implying that pathogen factors rather than host factors play an important role in modification of MGIR [209, 364]. Contradictory results have been obtained in different study [196] when a strain of* S. uberis* used to induce CM* in vivo* failed to cause a change in the mRNA levels of the immune-related genes by bovine MECs in culture, suggesting that the expression of immune-related genes by MECs may be initiated by host factors and not* S. uberis*. However, in the same study, challenging bovine MECs with different* S. uberis* strains resulted in an increase in the mRNA expression of a subset of the immune-related genes measured. Also, MGIRs towards different strains of* S. uberis* isolated from different IMI cases of different intensities varied. Expression of IL-1*β* and IL-8 from MECs* in vitro* has been shown to be greater with exposure to living and heat-inactivated* S. uberis* isolated from acute mastitis than* S. uberis* isolated from chronic mastitis [209]. More interestingly, a strain of* S. uberis* that induced acute mastitis* in vivo* caused twofold and fourfold higher expression of IL-8 and IL-1*β*, respectively, in isolated MECs* in vitro* than a strain isolated from a case of chronic mastitis [363]. Similar results were obtained in a separate study [209], indicating that the severity of mastitis induced by different* S. uberis* strains* in vivo* can be reflected at the level of the MGIR* in vitro*. In another* in vitro* study, heat-inactivated* S. uberis* did not trigger an IR from MECs, although inactivated* Staph. aureus* did, despite the fact that both bacteria are Gram-positive and contain LTA in their cell walls [330]. Continued to particularity of MGIR towards* S. uberis*, an emergence of* S. uberis*-specific bactericidal T-cells in the MGS of cows after infection or environmental exposure to* S. uberis *has been documented, suggesting that these specific cells may play a role in control of IMI caused by this bacterium [367].

To the best of our knowledge, no studies have been performed to assess the MGIR of bovines to the major contagious bacterium* S. agalactiae*. In a study of* S. agalactiae* IMI in mice [368], the IR manifested as a massive infiltration of MG by PMNs and the release of IL-1*β*, IL-6, and TNF-*α* in the first 72 h PI; these cytokine levels decreased concurrently with increased levels of IL-12 and IL-10.

Results obtained from different studies investigated the MGIR towards different mastitis pathogens, demonstrating the complexity of the MGIR to an infecting pathogen and indicating that a coordinated response exists between the resident, recruited, and inducible immune factors.

## 5. Future Perspectives

In recent years, there has been considerable expansion of our knowledge concerning host MG immune defence against bacterial infections. This defence involves sophisticated mechanisms for detecting various invading bacteria and combating them by the innate and acquired IRs. To improve dairy animal resistance against IMIs, further investigation concerning MG immunology should focus on the following: (1) enhancement of immune functions or at least the maintenance of these functions at normal levels under various lactating and nonlactating conditions, especially during periods of immune suppression; (2) clarifying the roles of specific mammary immune cells, primarily lymphocytes, and in particular the roles of NK cells and *γδ* cells, which are not fully defined; (3)* in vivo* and* in vitro* investigation of MGIRs against certain common bacteria in bovines, including* S. uberis*,* S. dysgalactiae*,* S. agalactiae*, coliforms other than* E. coli*, and CNS because most research studies concerning MGIRs have focused on* Staph. aureus* and* E. coli*, as most studies using other pathogens have involved experiments in ovines and focused on cytokine levels only without detailing the cellular responses; (4) clarifying the roles of certain chemokines as RANTES and cytokines such as IL-17, TGF, and CSF in MG, as well as LF effect against GPB because its role is not clearly understood; and (5) Changes of leukocytes population in MGs of ovines and caprines during IMIs.

## Figures and Tables

**Table 1:** Cellular elements in the BMG environment [1, 6, 7, 27, 28, 31, 32, 41, 43, 45, 63–65, 77, 80, 86, 336].

	Healthy MG	Mastitic MG
SCC	Usually lower than 1 × 10^5^ cells/mL milk. However, a SCC higher than 2 × 10^5^ cells/mL milk is considered to be a more practical distinguishing threshold for IMI.	SCC is greater than 2 × 10^5^ cells/mL milk according to severity of IMI; with severe IMIs, the SCC may reach 1 × 10^6^ cells/mL milk or more within a few hours.

Leukocytes	75% of SCC.	Dramatic increase occurs according to severity of IMI at early stages due to recruitment of immune cells from the marginal pool and bone marrow into the MG environment.

Macrophages	35–79% of total leukocytes in milk, constituting the predominant cell type.	9–32% of total leukocytes in milk.

Lymphocytes	10–28% of total leukocytes in milk. The proportions of T- and B-lymphocytes in milk are approximately 40–50% and 20–25%, respectively. *αβ* T-cells prevail and are predominantly CD8+ subset with memory characteristics (comprising approximately 50–60% of the T-lymphocyte population).	14–24% of total leukocytes in milk. CD4+ T-cells become the predominant activated phenotype in response to recognition of Ag-MHC class II complexes on Ag-presenting cells, such as B-cells or macrophages. In some circumstances, such as chronic *Staph. aureus* IMIs, CD8+ are predominantly recruited compared over CD4+ T-lymphocytes.

PMNs	3–26% of total leukocytes in milk.	The predominant cell type, constituting up to 90% of the total milk leukocytes or more. With chronic bacterial IMIs, PMNs also remain as the predominant cells, even for months.

**Table 2:** Sources and functions of important cytokines in the MG environment [111, 121, 134, 151, 162, 253, 256, 261, 275, 291–296, 298–303, 306, 309, 311–313, 318, 320–328, 369, 370].

Cytokine	Sources	Functions
IL-1	(i) Macrophages (ii) Lymphocytes (iii) Monocytes (iv) Endothelial cells (v) Fibroblasts	(i) A proinflammatory cytokine. Like TNF-*α*, it mediates generation of acute phase inflammatory and febrile responses and synthesis of APPS, mainly via IL-1*β*. (ii) Increases neutrophil recruitment to MG and enhances their phagocytic and bactericidal activities. (iii) Stimulate secretion of IL-1 itself and IL-6, IL-8, IL-12, and TNF-*α*.

IL-2	CD4+ cells, mainly Th1	(i) Regulates AIS via enhancing the proliferation of B-lymphocytes. (ii) Enhances cytotoxic and bactericidal activities of T-lymphocytes. (iii) Increases plasma cell numbers and activates NK cells.

IL-4	(i) CD4+, mainly Th2 (ii) CD8+/T-suppressors (iii) B-lymphocytes	(i) Contributes to regulation of IIS by regulating the differentiation of T-lymphocytes. It favours development of Th2 subsets. (ii) Exerts a clear inhibitory effect on IFN-*γ* production.

IL-6	(i) Macrophages (ii) Lymphocytes (iii) Monocytes (iv) Neutrophils (v) MG epithelium (vi) Endothelial cells	(i) A pleiotropic cytokine with both pro- and anti-inflammatory properties. (ii) Shares generation of febrile response and regulates APP synthesis. (iii) Favours the influx of monocytes into the MG. (iv) Induces B-cell differentiation and thus the corresponding Ig production and T-cell activation of neutrophils. (v) Exerts anti-inflammatory action by inhibiting expression of IL-1*β* and TNF-*α*.

IL-8	(i) Monocytes (ii) T-lymphocytes (iii) Macrophages (iv) PMNs (v) MG epithelium (vi) Endothelial cells	(i) Induces inflammatory response. It is a potent chemoattractant (chemokine) mainly for neutrophil migration into MG with a longer lasting effect and to lesser extent to T-lymphocytes. (ii) Induces neutrophil degranulation. (iii) Enhances microbicidal activities of PMNs and stimulates phagocytosis of opsonised particles.

IL-10	(i) Th2 cells (ii) B-lymphocytes (iii) Monocytes (iv) Eosinophils (v) Mast cells	(i) The main anti-inflammatory cytokine and a principle partner in inflammatory resolution. (ii) Prevents production of proinflammatory cytokines, chemokines, and eicosanoids by leukocytes and downregulates generation of all subtypes of T-helper cells. (iii) Impairs macrophages presentation of Ags to T-cells by downregulating MHC class II expression.

IL-12	(i) Macrophages (ii) B-lymphocytes (iii) Monocytes (iv) Neutrophils	(i) Acts as a mediator between IIS and AIS via regulating differentiation of T-lymphocytes. It favours the polarisation of CD4+ and CD8+ T-cells into Th1 and cytotoxic IFN-*γ* producers, respectively. (ii) Acts as a growth factor for activated NK cells and enhances their cytotoxic activities. (iii) By stimulating the production of IFN-*γ* by both T-cells and NK cells, it contributes to the activation of macrophages. (iv) Alters Ab responses by enhancing the production of Igs involved in opsonisation and the facilitation of cell-mediated responses, while impairing the production of Igs involved in mediating Th2 humoral IRs. (v) Like IFN-*γ*, it upregulates other cytokines as TNF-*α*, IL-8, and IL-10.

IL-17	Activated memory T-cells	(i) A proinflammatory cytokine, having a potential to modulate the MGIR to mastitis-causing pathogens. (ii) Appears to play an upstream role in T-cell-triggered inflammation by stimulating stromal cells to secrete other cytokines. (iii) Could play a role in the recruitment of neutrophils in the BMG during infection or immune-mediated inflammation through regulating IL-8. (iv) IL-17A, and to a lesser extent IL-17F, increase the expression of a number of genes encoding cytokines, chemokines, and proteins endowed with antibacterial activities. (v) Enhances expression of chemokines targeting neutrophils and mononuclear leucocytes. (vi) Enhances production of IL-6, IL-8, and Gro-*α* and the expression of inflammatory cytokines TNF-*α* and IL-1*β*.

G-CSF	(i) Fibroblasts (ii) Endothelial cells (iii) Macrophages (iv) T-lymphocytes	(i) Increases numbers of both blood and milk neutrophils. (ii) Increases phagocytosis and bactericidal activity of leukocytes.
M-CSF		(i) Potent macrophage chemoattractant. (ii) Regulates proliferation and differentiation of macrophages.
GM-CSF		(i) Enhances chemotaxis and bactericidal activities of neutrophils. (ii) Increases number of phagocytic cells and enhances their cytotoxic activities.

IFN-*γ*	(i) T-lymphocytes, mainly Th1 and CD8+/T cytotoxic subset (ii) NK cells (iii) Monocytes	(i) Similar to IL-12, it serves to bridge the innate and adaptive arms of the IS. (ii) Mediates activation and microbicidal activity of neutrophils and macrophages. (iii) Reverses suppressive effects of MG secretions. (iv) Induces production of IL-12 by different phagocytes. (v) Upregulates cell-surface MHC-I molecule expression, thus promoting the induction of cell-mediated immunity by increasing the likelihood of cytotoxic T-cell recognition of presented Ags. (vi) Upregulates MHC-II Ag presentation pathway and corresponding CD4+ T-cell activation.

TNF-*α*	(i) Macrophages (ii) Neutrophils (iii) MG epithelium	(i) The main cytokine produced during the early stage of infection. It enhances generation of febrile and acute phase inflammatory responses. (ii) Enhances neutrophil phagocytosis and bactericidal activity. (iii) Induces expression of adhesion molecules on endothelial cells. (iv) Stimulates secretion of IL-8 by different cells.

TGF-*α*	(i) Epithelial cells (ii) Fibroblasts (iii) Neutrophils (iv) Macrophages (v) Eosinophils	(i) A mediator of tissue repair and healing, MG epithelial proliferation, angiogenesis, and morphogenesis. (ii) Upregulates the production of prostaglandins and synergistically enhances the effects of IL-1*β* and TNF-*α*. (iii) Stimulates IL-8, prostaglandin-E2, and expression of antimicrobial peptides.
TGF-*β*		(i) Regulates ductal growth and patterning and alveolar development and functional differentiation. (ii) Exerts some pro- and anti-inflammatory properties. (iii) Induces extracellular matrix deposition and fibrosis after injury to the mammary epithelium, which contributes to the formation of scar tissue.

RANTES		(i) A member of the CC family of chemokines. It is involved in many immunoregulatory and inflammatory processes, though its exact roles during IMI are not clear. (ii) RANTES was found to be important for initiation of chemotaxis as well as maintenance of inflammation inside bovine MG.

**Table 3:** Igs in bovine colostrum and milk.

Igs (g/L)	Cow		Buffalo		Goat		References
	Milk	Colostrum	Milk	Colostrum	Milk	Colostrum	
IgA	0.05–0.14	1–6	0.01–0.03	0.18–0.57	0.03–0.08	0.9–2.4	[286, 371–376]
IgM	0.05–0.1	3–9	0.04	0.47–0.57	0.01–0.04	1.6–5.2	[286, 371–376]
IgG total	0.15–0.8	20–200	0.46–1.34	29.75–36.0	0.1–0.4	50–60	[286, 371–376]
IgG1	0.3–0.6	15–180	0.36–1.15	27.72–34.08	—	—	[286, 374–376]
IgG2	0.02–0.12	1–3	0.10–0.19	1.91–2.03	—	—	

**Table 4:** BMG immune response towards different mastitis-causative bacteria.

*Staph. aureus *	(i) Both SCC and leukocyte count increase (mainly PMNs), but leukocytes are not in a highly activated state [17, 86]. (ii) The CD8+ lymphocytes are preferentially recruited over CD4+ lymphocytes [78, 86]. (iii) Increase in the proportion of the B-lymphocytes in the total lymphocyte population, revealing development of humoral responses. No change was observed in the *γδ* T-lymphocyte subset [86]. (iv) Variable increases in IL-1*α*, IL-1*β*, IL-6, IL-12, TNF-*α*, IFN-*γ*, GM-CSF, GRO, and soluble CD14 [86, 114, 165, 329, 350]. (v) Depression of IL-2 and IL-4 levels [86, 350]. (vi) Chemoattractant IL-8 showed an undetectable change [165, 239] or slight increase [114, 329]. A lower level of C5a was observed [239]. (vii) Like IL-8, the anti-inflammatory cytokine IL-10 may exhibit an increase [86] or an undetectable increase [114]. (viii) The gene encoding proinflammatory cytokine IL-17A shows an increase in tissues of infected MG [17]. (ix) TGF-*β* showed no marked release [114]; meanwhile, a study by [135] showed increased TGF-*β* expression in MG. On the other hand, expression of TGF-*β* increased moderately in ductal tissue of MG and, on the contrary, moderately decreased in teat canal tissues [17].

*Staph. epidermidis *	In one comparative study by [354]: (i) Elevation of SCC, but lower than that observed with *Staph. simulans*. (ii) Elevation of IL-1*β*, IL-8, and TNF-*α* levels in milk. Systematically there are no changes in these cytokines, although the cows showed mild to moderate signs of CM.

*Staph. simulans *	(i) Increased leukocyte counts in milk with severe infiltration of MG tissues with mononuclear cells and neutrophils [358]. (ii) Increased IL-1*β*, TNF-*α*, and IL-8 levels in milk, more than that elicited by *Staph. epidermidis; *however, the levels of these cytokines remained unchanged systemically [354].

*S. uberis *	(i) The early host response to *S. uberis* infection is characterized by a relatively slow but massive influx of PMNL in the infected MG [336, 337, 365]. The percentage of lymphocytes expressing CD44 increased 12 h PI [343]. (ii) Increased leukocyte counts in milk and decreased counts of circulating leukocytes [336, 337]. (iii) Robust increase of chemoattractants such as IL-8 and C5a [336, 337]. (iv) Marked elevation of IL-1*β* and TNF-*α* proinflammatory cytokines, but the time of elevation differed among studies [209, 336, 337]. (v) Marked prolonged increase of IL-10, IL-12 [337], and IFN-*γ* [337, 367]. (vi) Elevation of sCD14 and LPS-BP, involved in host recognition of bacterial cell wall products; both of them remained elevated [337]. (vii) Investigation done to determine the most affected gene networks and pathways in MG tissue in response to an IMI with *S. uberis* has illustrated upregulation of several genes encoding IL6, TNF, IL8, IL10 and indicated that TNF had positive relationships with genes involved with immune system function (e.g., CD14, IL-8, IL-1*β*, and TLR2) [362]. (viii) Likewise, in another study to evaluate the transcriptional changes that occur in the MG after the onset of clinical *S. uberis* mastitis, an increase in mRNA expression of immune-related genes, complement component 3, IL-8, IFN-*γ*, IL-10, IL-1*β*, IL-6, TLR2, TNF-*α*, LF, LPS-BP, and oxidative stress-related genes metallothionein 1A and superoxide dimutase 2, has been confirmed [196].

*S. dysgalactiae *	(i) Increased leukocyte counts in milk, mainly PMNs. (ii) Increase of TLR4 expression and elevation of IL-1*β* and TNF-*α* [361].

*E. coli *	(i) Rapid and intense increase in milk SCC [165] including increases in leukocyte counts and intense PMN recruitment to MG [127, 165, 239]. (ii) Marked elevation of proinflammatory, regulatory, and anti-inflammatory cytokines including IL-1*β*, IL-6, IL-8, IL-10, IL-12, GM-CSF, IFN-*γ*, soluble CD14, GRO, C5a [127, 165, 307, 329], TGF-*α*, TGF-*β*1, and TGF-*β*2 [311]. (iii) The level of TNF-*α* varied among studies, revealing undetectable values [307, 338, 339] or marked rises [114, 127, 165, 239, 329, 350, 377].

*Serratia marcescens *	Studies regarding *S. marcescens *were almost done in comparison to *S. uberis* [337, 343] showing the following. (i) Systemic reaction peaking at 24 h PI in *S. marcescens*-infected cows and dropping thereafter, compared with 96 h PI in *S. uberis*-infected cows. (ii) Increased leukocyte counts in milk while circulating leukocyte counts decreased. (iii) Transient increase in chemoattractants such as IL-8 and C5a. (iv) Transient increase in IL-1*β* and TNF-*α* proinflammatory cytokine levels in milk. However, the peak of TNF-*α* was more rapid and stronger compared to *S. uberis*. Likewise, IL-12 and IFN-*γ* were transiently increased. (v) Small increase in the anti-inflammatory cytokine IL-10 at 18 h and then a return to the prechallenge level. (vi) Transient increase of sCD14 and LPS-BP followed by a return to prechallenge levels. (vii) Lymphocytes expressing either CD62L or CD11a showed a marked increase 12 h PI.

*Klebsiella pneumoniae *	One study [275] showed the following. (i) Increased leukocyte counts in milk, mainly PMNs. (ii) Marked rapid increase in IL-8 and C5a chemoattractants after challenge with pathogen and IL-1*β*, TNF-*α*, IL-12, and IFN-*γ* proinflammatory cytokines. (iii) Marked increase in anti-inflammatory cytokine IL-10. (iv) Increase of LPS-BP and sCD14.

*Pseudomonas aeruginosa *	One study [37] showed the following. (i) Increase in SCC and leukocyte counts in milk for more than 3 weeks. (ii) Early increased levels of IL-8, IL-10, IL-12, and TNF-*α*. In contrast, IL-1*β*, IFN-*γ*, TGF-*α*, TGF-*β*1, TGF-*β*2, sCD14, LPS-BP, and C5a levels were elevated for sustained periods of >48 h.

## Abbreviations

MG(s): Mammary gland(s)
BMG(s): Bovine mammary gland(s)
CNS: Coagulase-negative staphylococci
IMI: Intramammary inflammation
CM: Clinical mastitis
SCM: Subclinical mastitis
IR(s): Immune response(s)
MGIR: Mammary gland immune response
IIR: Innate immune response
IIS: Innate immune system
AIS: Adaptive immune system
SCC: Somatic cell count
Abs: Antibodies
Ag: Antigen
MHC: Major histocompatibility complex
APP: Acute phase protein
APR: Acute phase response
CS: Complement system
CSF: Colony stimulating factor
FAs: Fatty acids
G-CSF: Granulocyte CSF
GM-CSF: Granulocyte-macrophage CSF
GRO-*α*: Growth related oncogene-*α*
IFN-*γ*: Interferon-*γ*
Igs: Immunoglobulins
IL(s): Interleukin(s)
rBOIL: Recombinant bovine interleukin
LPS-BP: LPS binding protein
LF: Lactoferrin
LPS: Lipopolysaccharides
LTA: Lipoteichoic acid
PG: Peptidoglycans
LZs: Lysozymes
M-CSF: Macrophage CSF
MECs: Mammary epithelial cells
NK: Natural killer cells
PAF: Platelet-activating factor
LAP: Lingual antimicrobial peptide
TAP: Tracheal antimicrobial peptide
PAMPs: Pathogen-associated molecular patterns
PMNs: Polymorphonuclear neutrophils
PR: Pathogen recognition
PRRs: Pathogen recognition receptors
ROS: Reactive oxygen species
TLR: Toll-like receptors
TNF(s): Tumour necrosis factor(s)
PI: Postinfection.
